# Revisiting the role of structural connectivity-based parcellation in thalamic nuclei segmentation: Benchmarking against recent state-of-the-art methods

**DOI:** 10.1371/journal.pone.0351431

**Published:** 2026-06-15

**Authors:** Daniel H. Nguyen, Debottama Das, Ali Bilgin, Dianne Patterson, Matthew Hook, Chris Butson, Alberto Cacciola, Vinod Kumar Jangir, Manojkumar Saranathan

**Affiliations:** 1 University of Massachusetts Chan Medical School, Worcester, Massachusetts, United States of America; 2 Department of Electrical and Computer Engineering, University of Arizona, Tucson, Arizona, United States of America; 3 University of Arizona at Tucson, Tucson, Arizona, United States of America; 4 Department of Neurology, University of Florida, GainesvilleFlorida, United States of America; 5 Department of Biomedical Sciences, Humanitas University, Via Rita Levi Montalcini, Pieve Emanuele, Milan, Italy; 6 IRCCS Humanitas Research Hospital, Via Manzoni, Rozzano, Milan, Italy; 7 Max Planck Institute for Biological Cybernetics, Tuebingen, Germany; 8 Department of Radiology, University of Massachusetts Chan Medical School, Worcester, Massachusetts, United States of America; University of Minnesota, UNITED STATES OF AMERICA

## Abstract

Leveraging diffusion tractography, connectivity-based parcellation (CBP) is one of the oldest methods for thalamic nuclei segmentation. The goal of this work was to reassess CBP using higher spatial resolution diffusion MRI data and reconstruction algorithms, and to compare it with recent state-of-the-art methods for thalamic nuclei segmentation. Furthermore, these methods were systematically evaluated against three histological atlases and one functional MRI–based atlas to examine their relative anatomical similarities and differences. High resolution diffusion and T1-weighted MRI data from 67 healthy individuals in the Human Connectome Project Young Adult database were analyzed. CBP was performed using probabilistic tractography with cortical targets derived from combining labels of the Human Connectome Project Multi-Modal Parcellation 1.0 atlas into 8, 11, and 23 regions. Results were compared against three recent methods: orientation distribution function clustering (ODF), track density imaging (TDI), and structural MRI-based segmentation. Group level analyses were conducted in the Montreal Neurological Institute space, and Dice overlap coefficients were calculated using four atlases (three histological, one functional). CBP results using newer data and methods were still remarkably similar to the original CBP parcellation results. Across atlases, a consistent hierarchy was observed: HIPS-THOMAS performed best, followed by TDI, ODF, and CBP (Kendall’s W = 1.00, p = 0.007). Histological atlases showed strong mutual agreement (Pearson *r* = 0.71–0.85), whereas the Zhang atlas demonstrated lower concordance (Pearson *r* = 0.51–0.63). Despite methodological advances, CBP remains constrained in its ability to delineate thalamic nuclei with histological accuracy. By contrast, structural and diffusion microstructural approaches provided better nuclear localization. These findings highlight the need for hybrid workflows that integrate structural and diffusion-based information to enable more reliable thalamic segmentation for neuroscience research.

## Introduction

The thalamus is a subcortical brain structure that plays a central role in relaying and integrating sensory, motor, and cognitive information between cortical, subcortical, and cerebellar regions. It is functionally organized into individual thalamic nuclei, each characterized by unique connectivity patterns to specific brain regions. These nuclei are highly relevant in a variety of diseases, with structural and functional alterations often linked to specific clinical scenarios. For instance, the ventral posterolateral nucleus of the thalamus relays somatosensory information [1] and has been associated with neuropathic pain and nociception [2], whereas the mediodorsal nucleus is associated with executive function and has been implicated in chronic neurodegenerative diseases, including Alzheimer’s disease [3,4], multiple sclerosis [5], and frontotemporal dementia [6]. The pulvinar nucleus, with its widespread connections to visual, auditory, somatosensory, parietal, temporal, and occipital cortices, plays a critical role in higher-order sensory integration and multimodal processing [7–10]. Dysfunction of the mesial pulvinar has been linked to temporal lobe epilepsy, where seizures propagate through its extensive cortical connections [11]. Due to the involvement of thalamic nuclei in several neurodegenerative diseases, fast, automated segmentation of thalamic nuclei is critical to probe the role of these structures from large public databases such as Alzheimer’s disease neuroimaging initiative (ADNI). Functionally important nuclei, like the ventral intermediate nucleus (Vim), are therapeutic targets for deep brain stimulation (DBS) and MR-guided focused ultrasound (MRgFUS) in essential tremor [12], while others, such as the medial pulvinar, anteroventral, and centromedian, are being investigated for epilepsy DBS [9,13,14].

Over the past two decades, several methods for direct and precise delineation of thalamic nuclei have been proposed. Connectivity-based parcellation (CBP) emerged with the advent of diffusion-weighted imaging (DWI), leveraging connectivity patterns to assign thalamic voxels to specific cortical regions of the brain [15]. Behrens et. al. (2003) pioneered this approach, segmenting the thalamus into seven different cortical connectivity-based parcels [15]. Since their seminal work, other studies have confirmed the reproducibility of connectivity-based parcellation (CBP) while refining its methodology [16]. For instance, a 2010 study demonstrated that thalamic CBP is highly reproducible when using broad cortical targets, but reproducibility decreases substantially with finer parcellations involving 31 cortical targets [17]. CBP has also been extended to the subthalamic nucleus [18–23] while another study demonstrated distinct subdivisions of the MD nucleus, that aligned well with known functional and anatomical territories [24]. Additionally, CBP has also been used to parcellate the GPi [23,25–27]. Finally, CBP (along with functional MRI information) is the main methodological driver in the more recent Human Brainnetome Atlas [28], which has been used in many studies examining at thalamic nuclei involvement in bulimia nervosa, chronic insomnia, and bipolar disorder [29–31].

Other approaches based on diffusion MRI at a local level without requiring long range tractography and cortical target definitions have been proposed. The most successful of them use diffusion tensor imaging (DTI) [32] and involve different methods to cluster the primary angular orientation of the diffusion tensor [33–35]. Another study used spherical harmonic representations of orientation distribution function (ODFs) to differentiate intra-thalamic microstructures [36]. ODF-based parcellation was demonstrated to be much more stable and reproducible compared to angular direction approaches. Basile et al. (2021) introduced short-tracks track-density imaging (TDI) providing maps with similarities to that of a histological thalamic atlas [37]. The utilization of resting-state functional magnetic resonance imaging (fMRI) emerged shortly after the introduction of CBP, identifying functional subdivisions of the thalamus through temporal correlations in spontaneous BOLD signal fluctuations between thalamic voxels and the cortical network [38]. Early work by Zhang et al. (2008) demonstrated strong functional specialization of individual thalamic voxels [39], which was later refined by using temporally independent thalamocortical states [38] and instantaneous connectivity parcellation to delineate functionally distinct subregions [40]. Lastly, other structural and hybrid connectivity-based approaches have also been proposed, including 3D edge [41], and Diffusion MRI for Anatomical Nuclei Imaging (DiMANI) [42],

The primary reason for the dominance of diffusion-based, and to an extent, fMRI-based methods for thalamic nuclei segmentation until recently is the poor intrathalamic contrast of structural T1 and T2 weighted MRI. In the last 5 years, several advances have resulted in the resurgence of structural MRI-based thalamic nuclei segmentation. A probabilistic atlas-based Bayesian segmentation from ex-vivo histology and in-vivo Magnetization Prepared Rapid Gradient Recalled Echo (MP-RAGE) T1 weighted MRI has been proposed and is part of Freesurfer [43]. Specialized sequences such as Fast Gray Matter Acquisition T1 Inversion Recovery (FGATIR) [44], white-matter-nulled MP-RAGE (WMnMPRage) [45], and Quantitative Susceptibility Mapping [46,47] have been proposed for improved visualization of thalamic nuclei [48,49]. These have gone hand-in-hand with segmentation methods such as the Thalamus Optimized Multi-Atlas Segmentation (THOMAS) technique [50], which leverages the improved contrast of WMnMPRage [45] and its recent extension Histogram-based polynomial synthesis (HIPS)-THOMAS, which synthesizes white-matter-nulled images from conventional T1-weighted MRI prior to segmentation [51]. However, to our knowledge, no studies have comprehensively compared and evaluated CBP methods against other recent parcellation methods in the same cohort of subjects.

In this study, we sought to revisit CBP, leveraging state-of-the-art high resolution diffusion MRI data, advanced diffusion processing methods such as multi-shell multi-tissue constrained spherical deconvolution (MSMT-CSD) that account for tissue properties and handle crossing fibers, and cortical parcellations derived from sophisticated atlases such as the Glasser Human Connectome Project – Multi-Modal Parcellation, version 1.0 (henceforth referred to as just Glasser atlas) [52]. Our first aim was to assess the performance of “modern” CBP, segmenting the thalamus into parcellations based on cortical connectivity, with the objective of achieving parcellations that achieve closer resemblance to histological-based atlases than the original implementation. We then compared CBP-derived parcellations with three state-of-the-art methods leveraging diffusion microstructure, short-range structural connectivity, and structural MRI. Finally, we compared the four methods against four atlases – three histology-based and one functional MRI-based atlas – to assess their relative anatomical similarity and differences.

## Materials and methods

### Participants and imaging data

No new data was collected for this project. Data was obtained from the Human Connectome Project (HCP) Young Adult (YA) 1200 Subjects database [53], which was publicly released on March 1, 2017. The authors of this manuscript accessed the data on August 27, 2021, and did not have access to information that could identify individual participants during or after data collection. The original study was approved by the Washington University Institutional Review Board (IRB number 201204036), and written informed consent was obtained from participants. A subset of n = 67 healthy young adults (ages 22–35) was selected based on the availability of both structural T1-weighted and high-resolution diffusion MRI data. All participants provided informed consent, and the Institutional Review Board approved the HCP protocols.

### MRI acquisition

**Fig 1:**
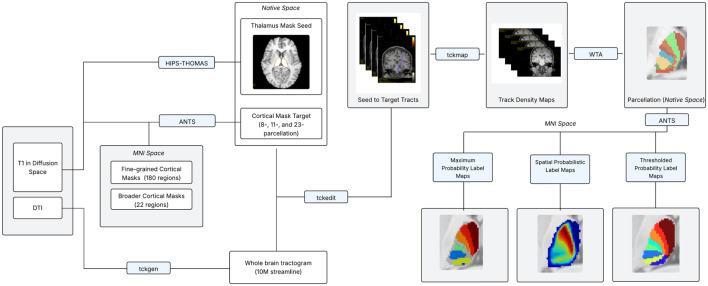
Workflow for Generating CBP of HCP-YA Subjects in HCP-YA subjects using HIPS-THOMAS, Glasser atlas, and MRtrix3-based probabilistic tractography.

A schematic of the entire workflow is shown in **Fig 1**. Structural T1-weighted images (HCP-YA) were acquired using a 3D MPRAGE sequence with 0.7 mm isotropic resolution (TR = 2400 ms, TE = 2.14 ms, TI = 1000 ms, flip angle = 8°) [53]. High-resolution diffusion MRI data (HCP-YA) were acquired using a spin-echo EPI sequence with 1.25 mm isotropic resolution, sampling three shells (b = 1000, 2000, 3000 s/mm²) across 270 diffusion directions, plus 18 b = 0 volumes. Acquisition was performed with both right-to-left and left-to-right phase encoding to enable correction for susceptibility-induced distortions [54,55].

### Image preprocessing

Structural MRI images were preprocessed using standard methods, including skull-stripping and bias-field correction. T1-weighted images were subsequently coregistered to diffusion space using Advanced Normalization Tools (ANTs) affine registration. DWI data underwent standard preprocessing using MRtrix3, including denoising, eddy-current and motion correction, bias field correction, and normalization of diffusion-weighted signal intensity.

### Cortical parcellation schemes

Cortical regions were delineated using the volumetric version of the Glasser atlas, available at https://osf.io/azup8, comprising 180 cortical parcels per hemisphere called “areas”. From these 180 “areas”, these can be further grouped into 22 larger “regions”, each comprising different functional characteristics as described in Glasser et al [52]. Subject-specific T1-weighted images, which have already been coregistered to diffusion space, were nonlinearly registered to the MNI152 (ICBM 2009a Nonlinear Asymmetric) template using ANTs (*antsRegistrationSyNQuick.sh*) with a symmetric normalization (SyN) transformation, producing both an affine matrix and nonlinear warp fields. These transformations were inverted to project the Glasser atlases (22- and 180-region) into each subject’s native T1/diffusion space using *antsApplyTransforms* with nearest-neighbor interpolation, yielding subject-space cortical parcellations to be used for the cortical target ROI. All spatial registrations were manually reviewed for each subject to confirm appropriate anatomical alignment, and no failed or clearly suboptimal registrations were identified among the subjects included in the final analysis. The 22- and 180-region atlases were used to create 3 cortical parcellation schemes (henceforth referred to as 8-parcellation, 11-parcellation, and 23-parcellation). The 8-parcellation essentially follows the same cortical scheme as Behrens et al. [15], with the addition of the frontal region. The 23-parcellation is derived from the 22-region atlas, with a split of the region that is defined as “Somatosensory and Motor Cortex” into separate somatosensory and motor cortical regions. Finally, the 11-parcellation is a middle ground between these two parcellation schemes, subdividing the visual cortex into a primary “visual” region and a “visual stream” region, separating the cingulate tract and posterior opercular regions, and introducing an auditory ROI derived from the temporal cortex. Details of each of these parcellation schemes are presented in **Table 1** and visualized in **S1 Fig**.

**Table 1:** Glasser atlas area division for each parcellation scheme.

8-Parcellation	Temporal: EC, PreS, H, PeEc, PHA2, TF, TGd, TGv, PHA1, PHA3, TE1a, TE1p, TE2a, TE2p, TE1m, PHT, H_L Parietal: PSL, STV, TPOJ1, TPOJ2, TPOJ3, 7Pm, 7AL, 7Am, 7Pl, 7PC, LIPv, VIP, MIP, AIP, LIPd, PFt, AIP, PFop, PF, PFm, PGi, PGs, PGp, IP2, IP1, IP0 Motor: 4 Sensory: 3b, 1, 2, 3a Visual: V1, V2, V3, V4, V6, V3A, V7, V3B, IPS1, DVT, V6A, V8, PIT, FFC, VMV1, VMV2, VMV3, VVC, MST, LO1, LO2, MT, V4t, FST, V3CD, LO3, PH Premotor: FEF, PEF, 55b, 6d, 6v, 6a, 6r Prefrontal: 47m, 10pp, 10d, 10r, p10p, 47s, OFC, 13l, 11l, 44, 45, 47l, a47r, 6r, IFJa, IFJp, IFSp, IFSa, p47r, SFL, 8Av, 8Ad, 8BL, 9p, 8C, p9-46v, 46, a9-46v, 9-46d, 9a, i6-8, s6-8 Frontal: p24pr, 33pr, a24pr, p32pr, a24, d32, 8BM, p32, 10r, 9m, 10v, 25, s32, pOFC, a32pr, p24
11-Parcellation	**Visual**: V1 **Visual Streams:** V2, V3, V4, V6, V3A, V7, V3B, IPS1, DVT, V6A, V8, PIT, FFC, VMV1, VMV2, VMV3, VVC, MST, LO1, LO2, MT, V4t, FST, V3CD, LO3, PH, TPOJ3 **Motor**: 4 **Sensory**: 3b, 1, 2, 3a **Cingulate Tract**: 5m, 5mv, 23c, 5L, 24dd, 24dv, SCEF, 6mp, 6ma, RSC, POS2, PCV, 7m, POS1, 23d, v23ab, d23ab, 31pv, ProS, DVT, 31pd, 31a, p24pr, 33pr, a24pr, p32pr, a24, d32, 8BM, p32, 10r, 9m, 10v, 25, s32, pOFC, a32pr, p24 **Premotor**: FEF, PEF, 55b, 6d, 6v, 6a, 6r **Posterior Opercular**: 43, OP4, OP1, OP2–3, FOP1 **Prefrontal**: 47m, 10pp, 10d, 10r, p10p, 47s, OFC, 13l, 11l, 44, 45, 47l, a47r, 6r, IFJa, IFJp, IFSp, IFSa, p47r, SFL, 8Av, 8Ad, 8BL, 9p, 8C, p9-46v, 46, a9-46v, 9-46d, 9a, i6-8, s6-8 **Parietal**: PSL, STV, TPOJ1, TPOJ2, TPOJ3, 7Pm, 7AL, 7Am, 7Pl, 7PC, LIPv, VIP, MIP, AIP, LIPd, PFt, AIP, PFop, PF, PFm, PGi, PGs, PGp, IP2, IP1, IP0, PSL, STV, TPOJ2 **Temporal**: EC, PreS, H, PeEc, PHA2, TF, TGd, TGv, PHA1, PHA3, TE1a, TE1p, TE2a, TE2p, TE1m, PHT, TPOJ1 **Auditory**: A1, 52, RI, PFcm, PBelt, MBelt, LBelt, TA2, STGa, A5, STSda, STSdp, STSvp, STSva, A4, PoI2, MI, Pir, AVI, AAIC, FOP3, FOP2, FOP4, FOP5, PI, PoI1, Ig
23-Parcellation	**Primary Visual: V1** **Early Visual**: V2, V3, V4 **Dorsal Stream Visual**: V6, V3A, V7, V3B, IPS1, DVT, V6A **Ventral Stream Visual:** V8, PIT, FFC, VMV1, VMV2, VMV3, VVC **MT+ Complex and Neighboring Visual Areas**: MST, LO1, LO2, MT, V4t, FST, V3CD, LO3, PH**Motor:** 4 **Somatosensory**: 3b, 1, 2, 3a **Premotor**: FEF, PEF, 55b, 6d, 6v, 6a, 6r **Posterior Cingulate**: RSC, POS2, PCV, 7m, POS1, 23d, v23ab, d23ab, 31pv, ProS, DVT, 31pd, 31a **Paracentral Lobular and Mid Cingulate**: 5m, 5mv, 23c, 5L, 24dd, 24dv, SCEF, 6mp, 6ma **Superior Parietal**: 7Pm, 7AL, 7Am, 7Pl, 7PC, LIPv, VIP, MIP, AIP, LIPd **Anterior Cingulate and Medial Prefrontal**: p24pr, 33pr, a24pr, p32pr, a24, d32, 8BM, p32, 10r, 9m, 10v, 25, s32, pOFC, a32pr, p24 **Dorsolateral Prefrontal**: SFL, 8Av, 8Ad, 8BL, 9p, 8C, p9-46v, 46, a9-46v, 9-46d, 9a, i6-8, s6-8 **Inferior Frontal**: 44, 45, 47l, a47r, 6r, IFJa, IFJp, IFSp, IFSa, p47r **Orbital and Polar Frontal**: 47m, 10pp, 10d, 10r, p10p, 47s, OFC, 13l, 11l **Posterior Opercular**: 43, OP4, OP1, OP2–3, FOP1 **Early Auditory**: A1, 52, RI, PFcm, PBelt, MBelt, LBelt **Auditory Association**: TA2, STGa, A5, STSda, STSdp, STSvp, STSva, A4 **Insular and Fontal Opercular**: PoI2, MI, Pir, AVI, AAIC, FOP3, FOP2, FOP4, FOP5, PI, PoI1, Ig **Medial Temporal**: EC, PreS, H, PeEc, PHA2, TF, TGd, TGv, PHA1, PHA3 **Lateral Temporal**: TE1a, TE1p, TE2a, TE2p, TE1m, PHT**Temporo-Parieto-Occipital Junction**: PSL, STV, TPOJ1, TPOJ2, TPOJ3 **Inferior Parietal**: PFt, AIP, PFop, PF, PFm, PGi, PGs, PGp, IP2, IP1, IP0

### Connectivity-based parcellation

Probabilistic tractography was conducted using *MRtrix3* software to estimate structural connectivity between the whole thalamus and cortical regions. Whole brain tractograms consisting of ten million streamlines per subject were generated for each of the n = 67 HCP-YA subjects. Streamlines connecting the *whole* thalamus (segmented using HIPS-THOMAS, see description below) and specific cortical regions (defined by the Glasser atlas) were filtered using MRtrix’s *tckedit* function with default parameters, employing each of the masks as inclusion regions of interest (ROIs). We assessed whole thalamus connectivity using cortical parcellations defined by the 8-parcellation, 11-parcellation, and 23-parcellation. Track density maps (TDMs) were computed from the filtered streamline datasets. Each TDM was normalized by using the thalamus mask as a spatial reference template to ensure consistent comparisons across subjects. A Winner-Take-All (WTA) approach was applied to assign each voxel within the thalamic nuclei to the cortical region demonstrating the highest normalized connectivity. This resulted in subject-specific, connectivity-driven thalamic segmentation maps (parcellations) in native space, one for each of the three parcellation schemes.

### Comparative analyses

To compare CBP with state-of-the-art methods for thalamic nuclei segmentation, comparative analyses were conducted between CBP-derived segmentations, specifically the 8-parcellation, to ODF-based k-means clustering, TDI-based parcellation, as well as HIPS-THOMAS structural MRI segmentation. These output maps from these methods would then be visually compared with the Morel atlas, which was selected as a representative histological reference due to its widespread use and its foundational role in commonly implemented segmentation tools such as THOMAS and FreeSurfer [43]. This serves as a well-established anatomical anchor to illustrate systematic differences in organizational principles (probabilistic connectivity gradients versus cytoarchitectonically defined nuclei). All results were visualized using standardized slice orientations in MNI space and consistent color-coding schemes were used as much as possible to facilitate direct comparison across methods. The three methods used for comparisons are briefly described below.

### ODF-clustering

Thalamic parcellation using ODF clustering was performed following the framework described by Battistella et al. (2017) [36]. Briefly, diffusion MRI data were first modeled using a spherical harmonic (SH) representation of the orientation distribution functions to provide full angular characterization of the diffusion process within each voxel. K-means clustering, initialized in a data-driven manner, was then applied to group thalamic voxels using a weighted average of Euclidean distance of voxel positions and SH coefficients as a distance metric, yielding seven reproducible thalamic nuclear clusters. The whole thalamus mask from HIPS-THOMAS was used instead of Freesurfer as in Battistella et al. (2017) but hyperparameters were kept identical.

### Track density imaging

A probabilistic version of a TDI thalamic atlas based on manual parcellation of individual TDI from 6 HCP-YA subjects (3 males, 3 females) [37] was used, freely available at https://github.com/BrainMappingLab/TDI-derived-Thalamic-Atlas. Briefly, for each subject, a manually defined bounding-box ROI encompassing the diencephalon was drawn using anatomical landmarks to optimize streamline density and TDI contrast. Within this ROI, short-tract tractograms were generated using the iFOD2 algorithm and from these tractograms, two super-resolution short-track TDI (stTDI) maps (0.25 mm isotropic) were produced: (i) a directionally encoded color stTDI (DEC-stTDI) and (ii) an apparent fiber density–weighted stTDI (AFD-stTDI). Manual delineation of 13 thalamic nuclei (anterior, centromedian-parafascicular, habenula, lateral dorsal, lateral geniculate, mediodorsal-centrolateral, mediodorsal, medial geniculate, midline nuclei, pulvinar, ventral anterior, ventral lateral and ventral posterior) was performed on stTDI maps. Segmentation was performed in sagittal slices using DEC-stTDI maps (with AFD-stTDI overlays at ~80% opacity), guided by histological sections [56]. The resulting 3D volumes were refined in axial and coronal planes, median filtered, eroded to minimize boundary overlap, binarized, and resampled to 0.5 mm³ resolution.

### HIPS-THOMAS

For structural MRI based segmentation, HIPS-THOMAS [51], a variant of the original THOMAS [50] method that is optimized for T1-weighted MRI was used. It uses a simple polynomial algorithm to synthesize WMn-like images prior to THOMAS. THOMAS itself is a classic multi-atlas segmentation method that uses a multi-atlas of 20 WMn-MPRAGE datasets, each manually segmented using the Morel atlas as a guide. After warping them to native space using ANTs nonlinear registration, the 20 segmentations are combined into a single final segmentation using a joint label fusion algorithm which uses local image similarity to differentially weight the labels [57].

### Group-level analysis

Native space parcellation results were spatially transformed into a standardized MNI template (ICBM 2009a Nonlinear Asymmetric) space using ANTs nonlinear registration. These parcellations, now all in MNI space, were then stacked voxel-wise in MNI space to generate a single image. This was then analyzed using three approaches.

### Maximum probability label maps

To compute the maximum probability label at each voxel across subjects, a voxel-wise majority voting strategy was performed. For each voxel in the thalamus, the most frequently assigned parcel label across individuals was identified. This resulted in maximum probability label maps in which each voxel was labeled according to the most common assignment across the cohort.

### Spatial probabilistic label maps

Binary masks were generated for each label and averaged across subjects, yielding voxel-wise probability maps. Each probability map was multiplied by the corresponding label index, and the results were summed to create a single weighted image. This representation highlights dominant nuclei while also reflecting the degree of labeling uncertainty across subjects, which appears as fuzzy boundaries.

### Thresholded probability label maps

At each voxel, the frequency of each nucleus label across subjects was computed, producing probability maps. The voxel was assigned to the label with the highest probability, provided that the winning label exceeded a pre-determined (e.g., 25% or 50%) threshold. Voxels that failed to meet this criterion were reset to background (i.e., zero), unlike the Maximum Probability Label Maps, which contain labels for every voxel.

### Quantitative performance evaluation

For quantitative comparisons, Dice coefficients were also computed for the four methods (CBP, ODF, TDI, and HIPS-THOMAS) and compared against four atlases that served as reference (Morel Atlas [58], Allen Human Brain Atlas [59], Ilinsky Atlas [60], and Zhang Atlas [39]. No additional masking or reconciliation step was applied to enforce agreement in overall thalamic extent between each method and the atlas; voxels labeled as thalamus by one method but not present in the reference atlas (and vice versa) were treated as non-overlapping in the Dice coefficient calculation. Because of the differing number of nuclei/clusters between the 4 parcellation methods and the 4 atlases, an MxN matrix ***D*** was generated, where each element ***D****(i,j)* is the Dice coefficient between nucleus/cluster *i* of the parcellation and nucleus/cluster *j* of the atlas. By computing maximum Dice along columns or rows, a vector of Dice coefficients comprising all nuclei/clusters can be generated for consistent comparisons across atlases.

### Atlas concordance

To assess concordance across atlases, methods were first ranked within each atlas according to their average Dice performance across nuclei. For each nucleus, methods were assigned ordinal ranks from 1 (highest Dice overlap) to 4 (lowest Dice overlap). These nucleus-level ranks were then averaged within each atlas to generate a single mean rank per method for that atlas, producing a rank-order table with atlases treated as raters and methods as objects. Kendall’s coefficient of concordance (W) was subsequently computed from this rank matrix to quantify the degree of agreement in method performance across atlases.

To further assess consistency across atlases, correlation was computed between atlas-specific Dice profiles for each method. Specifically, a Dice vector comprising maximum Dice values of all nuclei/clusters described earlier was used to generate concordance between all 6 atlas pairs (e.g., Morel–Allen, Morel–Ilinsky, etc.) using the Pearson correlation coefficient. To ensure robustness, correlations were aggregated using both Fisher’s r-to-z transformation in addition to direct arithmetic means of Pearson coefficients.

## Results

**Fig 2:**
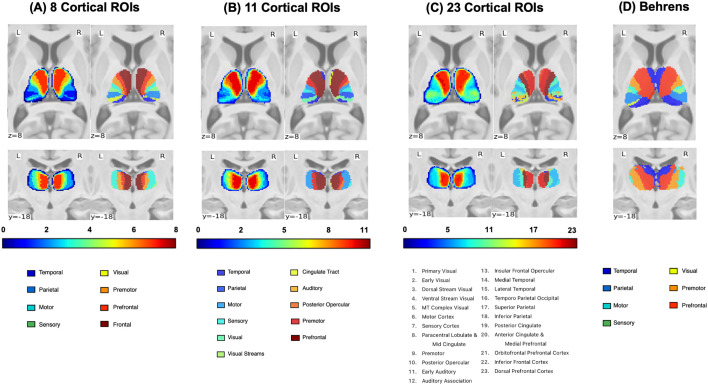
Cortical-based parcellation between Thalamus and 8/11/23 Cortical Regions of Interest. Results of CBP between the thalamus mask and 8-cortical regions **(A)**, 11-cortical regions (B) and 23-cortical regions (C) derived from the atlas of Glasser et al (2016) [52]. For the 8 regions, the 7 original cortical regions of Behrens et. al (2003) [15] were combined with the Frontal Region (dark red). For each of the 8-,11-, and 23-region parcellations, the spatial probabilistic label maps are shown on the left and the maximum probability label maps are shown on the right. All results are averaged from 67 subjects in MNI space.

Results of CBP using 8-, 11-, and 23-cortical regions of interest based on the Glasser atlas (**S1 Fig**.) are shown in **Fig 2**. with spatial probabilistic label maps and maximum probability label maps shown for each case (left and right columns respectively for each case). The spatial probabilistic label maps show stability/news based on the fuzziness of the borders while the maximum probability label maps depict each of the thalamic-cortical connections by a specific color, revealing the layered arrangement of parcels across axial and coronal slices. For reference, the parcellation map from Behrens et al. (2003) at 25% maximum probability is also shown, along with the 25% thresholded probability label maps for all three CBP schemes shown in **S2 Fig**.

### Thalamus connectivity with 8-parcellation

In the Behrens parcellation, the prefrontal and frontal cortex were represented as a single region (light red), whereas in our 8-parcellation this region is subdivided into prefrontal (light red) and frontal (dark red) components. Consistent with Behrens et al., we observed a distinct anterior-posterior layering pattern on the axial slice: premotor, motor, sensory, parietal, and visual cortical, a pattern that is also recapitulated in our 8-parcellation (**Fig 2A**, **S2A Fig**).

### Thalamus connectivity with 23-parcellation

As can be expected given the increase in thalamo-cortical connections, this parcellation scheme resulted in noisier parcellation patterns (e.g., auditory Label 11, **Fig 2C**, and posterior cingulate cortices Label 19, **Fig 2C**). After applying a 25% probability threshold, the posterior thalamic regions were largely absent (**S2C Fig**), suggesting that these voxels lack consistent connectivity patterns across subjects. This variability points to poor reproducibility of posterior thalamic labels in the 23-parcellation scheme.

### Thalamus connectivity with 11-parcellation

Given the relatively stable parcellations observed with the 8-parcellation (and concordance with the original Behrens atlas) and the noisiness observed in the 23-parcellation, we experimented with 11 cortical regions as a middle ground to balance anatomical details with stability/noise. The “frontal” region used in the 8-parcellation was reorganized. The results are shown in **Fig 2B**. The resulting 11-parcellation provides a somewhat more fine-grained thalamic map although some limitations persisted. Notably, connections with the auditory regions are significantly absent after the winner-takes-all parcellation, perhaps due to significantly poor streamline generation between the thalamus and auditory cortex.

### Comparisons between thalamic parcellation methods

**Fig 3:**
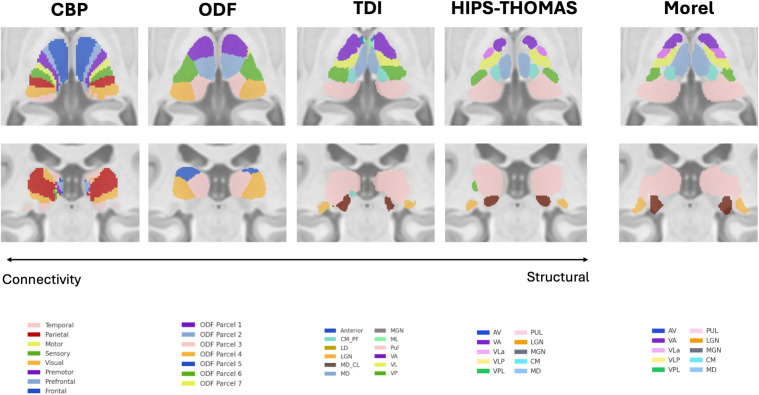
Comparison of CBP 8-parcellation maximum probability label maps with ODF clustering, TDI, and HIPS-THOMAS segmentations shown for comparable axial and coronal slices in MNI space (n = 67 subjects). The Morel atlas is shown on the far right for comparison.

Comparative axial and coronal views at the same slice level are shown for 8-parcellation CBP with the corresponding ODF clustering, TDI, and HIPS-THOMAS segmentation in MNI space **(Fig 3)**. The Morel atlas is also shown for reference. The methods showed distinct patterns depending on the basic characteristics each method relies on for parcellation. HIPS-THOMAS leverages the intrinsic contrast contours produced by the synthesized WMn contrast and is not surprisingly, closest to the Morel atlas, given the priors in THOMAS atlas were delineated using the Morel atlas as a reference (visual inspection). TDI which uses very short-range fibers and super resolution comes next, showing significant correspondence to the Morel atlas. ODF also uses diffusion microstructure but is at a lower resolution than TDI and produces clusters which comprise multiple nuclei in some cases (AV + VA for example) and splits some nuclei into adjacent clusters (pulvinar for example). Finally, CBP with its long-range connectivity looks farthest from the Morel architectonic parcellation as it is both dependent on functional information (specific cortical regions) and long-range connectivity. Interestingly, the subdivision of the pulvinar nucleus seen in the CBP clustering, with the lateral parts connecting to visual (occipital) cortex and medial parts connecting to the temporal cortex, is also seen in the ODF clustering.

### Quantitative performance evaluation

**Fig 4:**
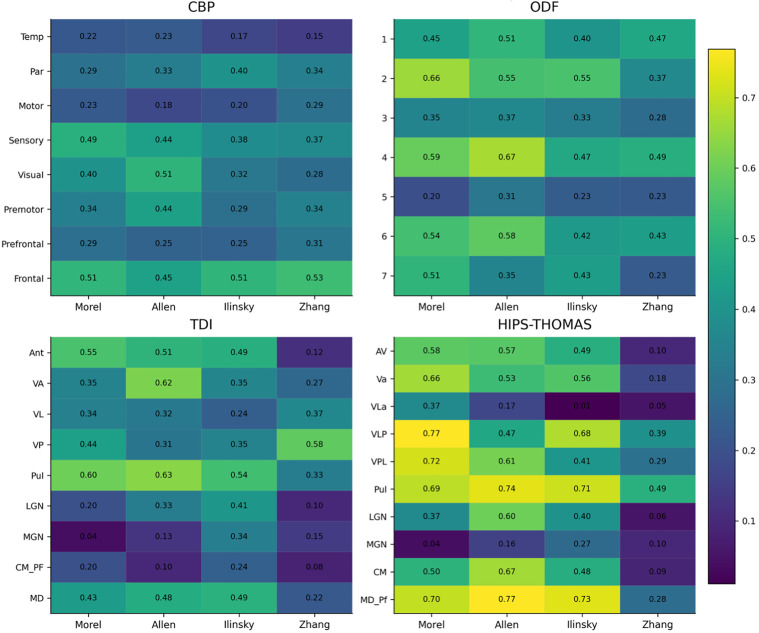
Maximum Dice coefficients between each parcel in 4 atlases (Morel, Allen Human Brain, Ilinsky, and Zhang) and its corresponding region in the CBP, ODF, TDI, and HIPS-THOMAS parcellations.

For quantitative comparison, Dice overlap scores were computed for all four methods (CBP, ODF, TDI, and HIPS-THOMAS) against the Morel atlas, which served as the primary histological reference (**Fig 4**). For each nucleus, the cluster or parcellation yielding the highest Dice coefficient was selected. A confusion matrix comparing each parcel against the Morel nuclei is shown in **S3 Fig**. The highest Dice values were observed in the sensory and frontal parcellations of the 8-parcel solution, both exceeding 0.50. ODF achieved the greatest Dice scores for Parcel 2 (Dice = 0.659) and Parcel 4 (Dice = 0.594), corresponding to the mediodorsal and pulvinar nuclei, respectively. TDI demonstrated the highest Dice values for the anterior (Dice = 0.550) and pulvinar (Dice = 0.603) parcellations. HIPS-THOMAS achieved the highest overall performance across parcellations, with Dice scores of 0.769 and 0.719 for the VLP and VPL nuclei, respectively.

**Fig 5:**
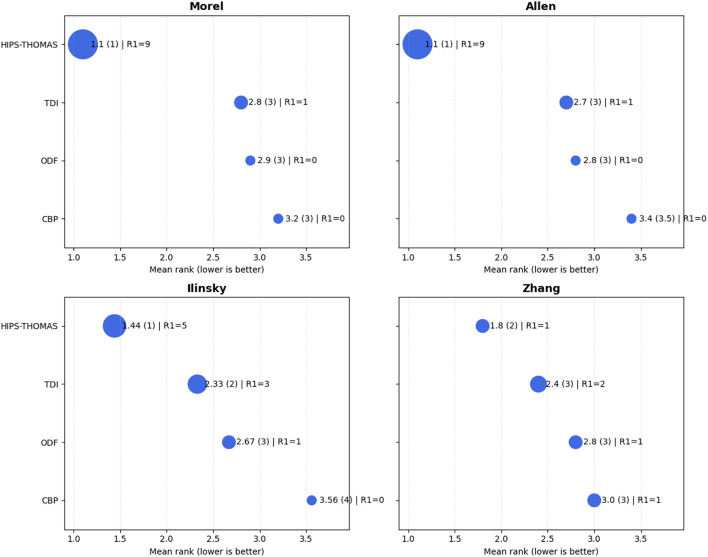
Method Rankings Across Nuclei by Atlas. For Mean Rank, lower mean and median suggests better ranking. With Kendall’s W testing, W = 1.00, p = 0.00738.

To determine whether these findings were specific to the Morel atlas or generalizable across reference frameworks, additional Dice comparisons were performed against the Allen Human Brain, Ilinsky, and Zhang atlases (**S4****–****S6 Figs**). The ontology mapping between atlas labels and method-specific parcels is shown in **S1 Table**. Across all the atlases (Morel, Allen Human Brain, Ilinsky, and Zhang), a consistent hierarchy emerged: HIPS-THOMAS demonstrated the highest Dice coefficients, followed by TDI, ODF, and CBP (**Fig 4**). Across nuclei, method rankings were highly concordant across atlases (Kendall’s W = 1.00, p = 0.00738; **Fig 5),** indicating a stable hierarchy of performance. For all atlases, HIPS-THOMAS consistently ranked first (lowest mean and median ranks and highest Rank-1 counts), followed by TDI, ODF, and CBP.

### Atlas concordance

Atlas-to-atlas Pearson correlation analysis (**S7 Fig**) first demonstrated a clear clustering pattern among reference frameworks. The classical histological atlases (Morel, Allen, Ilinsky) exhibited consistently strong positive correlations with one another across methods, indicating stable structural similarity in method-dependent Dice profiles. In contrast, correlations between the connectivity-based Zhang atlas and the histological atlases were systematically lower and more variable, suggesting reduced concordance with the classical atlas cluster. To further quantify this, mean correlations were computed across methods for each atlas pair (**Table 2**). The highest agreement was observed between Morel–Ilinsky (Fisher Z mean = 0.854; direct mean = 0.785) and Morel–Allen (Fisher Z mean = 0.770; direct mean = 0.768). In contrast, atlas pairs involving Zhang showed comparatively attenuated concordance, with Fisher Z mean correlations ranging from 0.603 to 0.631 (direct means: 0.512–0.615). Together, these findings confirm strong structural similarity among the histological Morel, Allen, and Ilinsky atlases, while indicating systematic divergence of the functional Zhang atlas from this histological atlases.

**Table 2:** Fisher Z-based mean correlations and direct arithmetic mean correlations for each atlas pair.

	Morel-Allen	Morel-Ilinsky	Allen-Ilinsky	Morel-Zhang	Zhang-Allen	Zhang-Ilinsky
**Fisher**	0.770	0.854	0.715	0.631	0.627	0.603
**Direct Mean Correlations**	0.768	0.785	0.712	0.615	0.530	0.512

## Discussion

The CBP method was introduced by Behrens et al. in 2003, nearly two decades ago. In this study, we revisited CBP using high-resolution diffusion MRI from the HCP-YA database, sophisticated diffusion MRI reconstruction methods that can handle crossing fibers, and cortical parcellations from the Glasser atlas, to ascertain if these improvements result in parcellation outcomes that are closer to the Morel cytoarchitectonic atlas. Further, we directly compared CBP results with recent best-in-class segmentation methods: ODF clustering, TDI, and HIPS-THOMAS, and evaluated their similarities/differences to the Morel atlas, in addition to two additional histological atlases and a functional MRI based atlas. To our knowledge, this is the first study benchmarking CBP against recent state-of-the-art methods. Our results demonstrate that, despite two decades of methodological progress, CBP showed little change in the resulting parcellation structure relative to its original implementation. It is important to note, however, that this study was designed as a practical evaluation of CBP’s limitations rather than as an effort to validate any single atlas against histology or to determine a universally “best” thalamic atlas. Comprehensive reconciliation across atlases remains methodologically unresolved. Prior expert-driven comparisons of histological atlases have demonstrated substantial variability in nuclear boundaries, nomenclature, and grouping conventions without clear consensus on one-to-one correspondence [61]. More recent community-level initiatives, including the ThAlamus Nuclei Neuroimaging GrOup (TANGO) roadmap [62], similarly highlight fundamental challenges in harmonizing atlases derived from different modalities, particularly when comparing cytoarchitectonic, structural MRI–based, and connectivity-based frameworks. Our results should therefore be interpreted within this broader context of cross-atlas variability.

Across our comparative analyses (**Fig 4**), other parcellation methods (ODF, TDI, HIPS-THOMAS) exhibited a closer spatial correspondence to the Morel atlas. Notably, ODF consistently outperforms CBP in terms of Dice overlap while also resolving the Pul nuclei into subdivisions that mirrored its known lateral (visual) and medial (temporal) connectivity and cytoarchitectonic profiles [7,11,63]. This finding likely reflects the fact that ODF clustering exploits intrinsic fiber orientation architecture rather than relying on predefined cortical targets, thereby preserving local topography and recovering boundaries that align more closely with cytoarchitectonic maps. TDI also benefits from high spatial resolution and directionality information, producing fine-grained patterns that are even closer to the histological maps. HIPS-THOMAS, however, achieved the highest Dice coefficients on average across all nuclei against all other methods, reaffirming its utility for anatomical delineation. It should also be noted that the THOMAS framework leverages structural MRI that has have very good intrathalamic contrast due to the white-matter-nulling and has priors manually annotated using the Morel histological atlas as a guide, thereby anchoring its segmentations to cytoarchitectonically defined thalamic boundaries. Most Dice coefficients with the exception of HIPS-THOMAS were low-to-moderate (<0.6), likely reflecting fundamental differences between ex vivo histological atlases and in vivo diffusion MRI–based segmentations, as well as boundary mismatches amplified in small nuclei. These values therefore represent relative spatial agreement rather than absolute failure, and CBPs should be interpreted as probabilistic approximations rather than precise cytoarchitectonic maps.

Importantly, our expanded cross-atlas analyses further demonstrate that this performance hierarchy is not dependent on a single reference atlas. Across all four atlases, method rankings were highly concordant (Kendall’s W = 1.00, p = 0.00738), indicating a stable and reproducible ordering of segmentation performance. HIPS-THOMAS consistently ranked first, followed by TDI, ODF, and CBP. This uniform ranking across heterogeneous atlases strengthens the interpretation that the observed differences reflect intrinsic methodological characteristics rather than a bias towards any particular atlas.

Now establishing that these are intrinsic methodological characteristics, CBP frequently produced parcels that did not align well with histological boundaries, and its performance was particularly limited in smaller nuclei. Like in the original Behrens atlas [15], subsequent variants by Traynor et al. [17], and in the most recent Brainnetome atlas [28], CBP produced a striped or “layer cake” pattern of parcels. This could be explained partly by the organized topographical projection of the thalamus to the cortex (e.g., mediodorsal to prefrontal cortex, ventro-lateral to motor/sensory areas, posterior to parietal/occipital areas and so on) which are continuous but forcibly discretized by the winner-take-all parcellation process. Thus, these bands should be interpreted not as nuclei but zones of dominant cortical connectivity. CBP parcels often show elongated medial–posterior gradients that do not align with the anterior–posterior nuclear organization seen in histological atlases such as Morel. This likely reflects the geometry of thalamocortical projection fibers, as tractography-based assignments are influenced by fiber entry and dispersion patterns rather than intrinsic cytoarchitectonic borders. Thus, CBP captures dominant connectivity gradients rather than discrete anatomical nuclei. This distinction is critical: CBP is fundamentally designed to model connectivity gradients, not to recover sharp cytoarchitectonic borders. The gradient nature of thalamocortical projections contrasts with the discrete nuclear boundaries defined histologically, making direct one-to-one correspondence inherently limited. These differences reflect divergent organizational principles rather than methodological failure. Another limitation in our implementation of the original CBP method of Behrens [15] is the exclusive use of cortical connectivity and lack of subcortical connections. Since we used a WTA strategy, this did not result in gaps or holes. However, in regions where subcortical inputs may dominate, such as ventral motor nuclei receiving predominantly pallidal and cerebellar projections, a cortically driven WTA approach may incompletely characterize the defining connectivity of that territory.

Other methodological characteristics may contribute to CBP’s limitations. First, the reliance on winner-take-all assignment converts marginal differences in neighboring track density maps into absolute labels, such that even minor fluctuations can flip voxel classification and destabilize parcellation boundaries (resulting in noise). Further, Clayden et al. (2019) have demonstrated that even when internal diffusion orientation information is randomly shuffled, voxel assignments remained consistent, suggesting that CBP parcellations may reflect extrinsic factors such as voxel proximity to major white matter pathways rather than internal thalamic organization [64]. Our findings also confirm that increasing the number of cortical targets, from 8 to 23 in this study, does not improve boundary definition, but instead exacerbates variability and noise, especially in posterior thalamic nuclei. This increased noise was observed in the 11-parcellation as well, albeit less pronounced. Taken together, this suggests that higher cortical target counts directly contribute to streamline tract variability without adding much more anatomical precision. Even with improved diffusion modeling via MSMT-CSD and higher spatial resolution (1.25 mm isotropic compared to the original 3 mm isotropic), histological validity appears constrained due to limitations in tractography and winner-take-all parcellation. However, these results are to be interpreted with caution since the Morel atlas is primarily a structural atlas with minimal functional information included. Its delineations were guided in part by immunohistochemical staining of calcium-binding proteins [58] (parvalbumin, calbindin, and calretinin), which reveal differential expression patterns across thalamic nuclei. Nonetheless, this approach still reflects structural cytoarchitecture rather than specific functional connectivity to cortical regions. CBP-based atlases may be more meaningful in studies analyzing structural and/or functional connectivity information than studies focusing on structural MRI data where schemes like HIPS-THOMAS might be more valuable.

Importantly, our results suggest that CBP may be less well suited for applications requiring precise nuclear localization, such as DBS or MRgFUS, where higher spatial accuracy is critical. One important consideration is reproducibility and operator-dependence. We employed a fully automated processing pipeline with out-of-the-box parameters across subjects for DTI processing to minimize these effects. However, this might still be suboptimal in a clinical environment where distortions from patient anatomy or subject motion might limit accuracy and require patient-specific adjustments which are impractical. For these applications, direct visualization enabled by recent advances in structural MRI spatial resolution would be more useful, especially as they are less immune to distortion and susceptibility artifacts that affect echo-planar imaging. This was also shown in a recent study showing the improved accuracy of anatomical-based targeting over DTI-based targeting using surgical outcomes as a metric of success [65]. In the thalamus, our findings indicate that structural methods like HIPS-THOMAS and diffusion microstructure–based approaches such as ODF clustering and TDI can provide complementary strengths and may in fact represent more reliable options in these contexts. Future work should therefore prioritize hybrid workflows that integrate these strategies, allowing investigators to balance connectivity, cytoarchitectonic, and microstructural information according to the needs of a given study.

Interventions such as DBS critically rely on accurate localization of desired nuclei, traditionally guided by a combination of standardized stereotactic coordinates, anatomical landmarks and/or by cytoarchitectonic atlases such as the Schaltenbrand Atlas [66], or, the Morel thalamic atlas [58], recent methods for automated segmentation such as THOMAS [67], or direct targeting [68]. However, atlas-based targeting has notable limitations, including reduced reliability/consistency in nucleus boundaries across patients [69]. In recent years, multimodal structural MRI techniques such as FGATIR, QSM, WMnMPRAGE, PD-weighted imaging, and related high-contrast structural sequences have substantially advanced direct visualization of thalamic nuclei for surgical targeting. In contemporary DBS and MRgFUS workflows, these approaches increasingly allow for patient-specific anatomical identification without sole reliance on atlas-based or diffusion-based indirect targeting. As such, connectivity-based parcellation may be less essential in the setting of direct surgical targeting than it was two decades ago. Our study, therefore, does not aim to replace or compete with these modern structural approaches. Rather, we sought to evaluate whether methodological advances in diffusion modeling and cortical parcellation meaningfully improve the histological validity of CBP itself. The finding that CBP remains structurally misaligned with cytoarchitectonic boundaries, even with high-resolution HCP data and advanced modeling, represents an important clarification for the field. It suggests that CBP captures dominant connectivity gradients rather than discrete nuclei and should be interpreted accordingly.

A final limitation of this study to raise is that all analyses were conducted in healthy young adults, whereas DBS and MRgFUS are typically performed in older patients with potentially altered thalamocortical connectivity. This concern is most relevant for CBP, which depends on long-range tractography, although similar questions may apply to other diffusion-based approaches. Prior studies have demonstrated that tractography-based parcellations can be obtained in individual DBS patients [21,22,26,42], and structural MRI–based methods such as THOMAS have also been applied in patient populations [70]. Nonetheless, future work is needed to directly evaluate the robustness of these methods in diseased cohorts. Additionally, CBP, as implemented here, is inherently constrained to cortical projection patterns and does not incorporate subcortical afferents from the brainstem, basal ganglia, or limbic structures, all of which contribute to thalamic organization [20]. As such, CBP cannot be expected to recover nuclei defined primarily by non-cortical connectivity. This limitation is intrinsic to the method rather than dataset-specific and further underscores that connectivity-based parcellations represent dominant cortical gradients rather than a comprehensive model of thalamic nuclear architecture.

In summary, our results indicate that, despite two decades of continued development, CBP retains some of the striped appearance of its original formulation. While it remains a widely used and valuable tool for broad thalamocortical mapping, our comparisons show that HIPS-THOMAS achieved the highest overall accuracy relative to histology-based atlases, while ODF clustering and TDI were most effective at delineating anatomically consistent boundaries for individual thalamic nuclei. These observations underscore the importance of carefully considering the strengths and limitations of different segmentation strategies, with approaches that directly model local fiber architecture holding promise for bridging in vivo imaging and histological precision.

## Supporting information

S1 Fig Cortical Regions defined by the Glasser Atlas used in each CBP Scheme. The following figure visualizes the cortical regions defined by the 8 parcellation (A), the 11 parcellation (B), and the 23 parcellation (C).
(TIFF)

S2 Fig 25% Threshold Probability Label Maps of CBP Between Thalamus and 8/11/23 Cortical Regions of Interest with Comparison to Behrens. “8 Cortical ROIs” depicts the 8 parcellation at 25% threshold probability. “11 Cortical ROIs” depicts the 11 parcellation at 25% threshold probability. “23 Cortical ROIs” depicts the 23 parcellation at a 25% threshold probability. The Behren parcellation in MNI space is also shown at a 25% threshold probability for comparison.
(TIFF)

S1 Table Ontology Table for Morel Atlas, Allen Human Brain Atlas, and Ilinsky Atlas. (DOCX)

S3 Fig Dice comparison between Morel Atlas versus all 4 methods. Dice scores were computed between each of the parcels in the Morel atlas and compared against each of the parcels in the 8 parcellation, ODF Clustering, TDI, and Structural (HIPS-THOMAS). Higher Dice signify greater overlap between the two parcels.
(TIFF)

S4 Fig Dice comparison between Allen Human Brain Atlas versus all 4 methods. Dice was conducted between each of the parcels in the Allen Human Brain atlas and compared against each of the parcels in the 8 parcellation, ODF Clustering, TDI, and Structural (HIPS-THOMAS). Higher Dice signify greater overlap between the two parcels.
(TIFF)

S5 Fig Dice comparison between Ilinsky Atlas versus all 4 methods. Dice was conducted between each of the parcels in the Ilinsky atlas and compared against each of the parcels in the 8 parcellation, ODF Clustering, TDI, and Structural (HIPS-THOMAS). Higher Dice signify greater overlap between the two parcels.
(TIFF)

S6 Fig Dice comparison between Zhang Atlas versus all 4 methods. Dice was conducted between each of the parcels in the Zhang atlas and compared against each of the parcels in the 8 parcellation, ODF Clustering, TDI, and Structural (HIPS-THOMAS). Higher Dice signify greater overlap between the two parcels.
(TIFF)

S7 Fig Atlas-to-Atlas Correlation of Dice Coefficients Across Parcellation Methods. Heatmap showing Pearson correlations (r) of Dice coefficients between atlas pairs for each parcellation method (CBP, ODF, TDI, HIPS-THOMAS).
(TIFF)
