# Supplementary figures and images for: Revisiting the role of structural connectivity-based parcellation in thalamic nuclei segmentation: Benchmarking against recent state-of-the-art methods
Source: PLoS One. 2026 Jun 15;21(6):e0351431. doi: 10.1371/journal.pone.0351431 (PMC13268177; doi:10.1371/journal.pone.0351431)

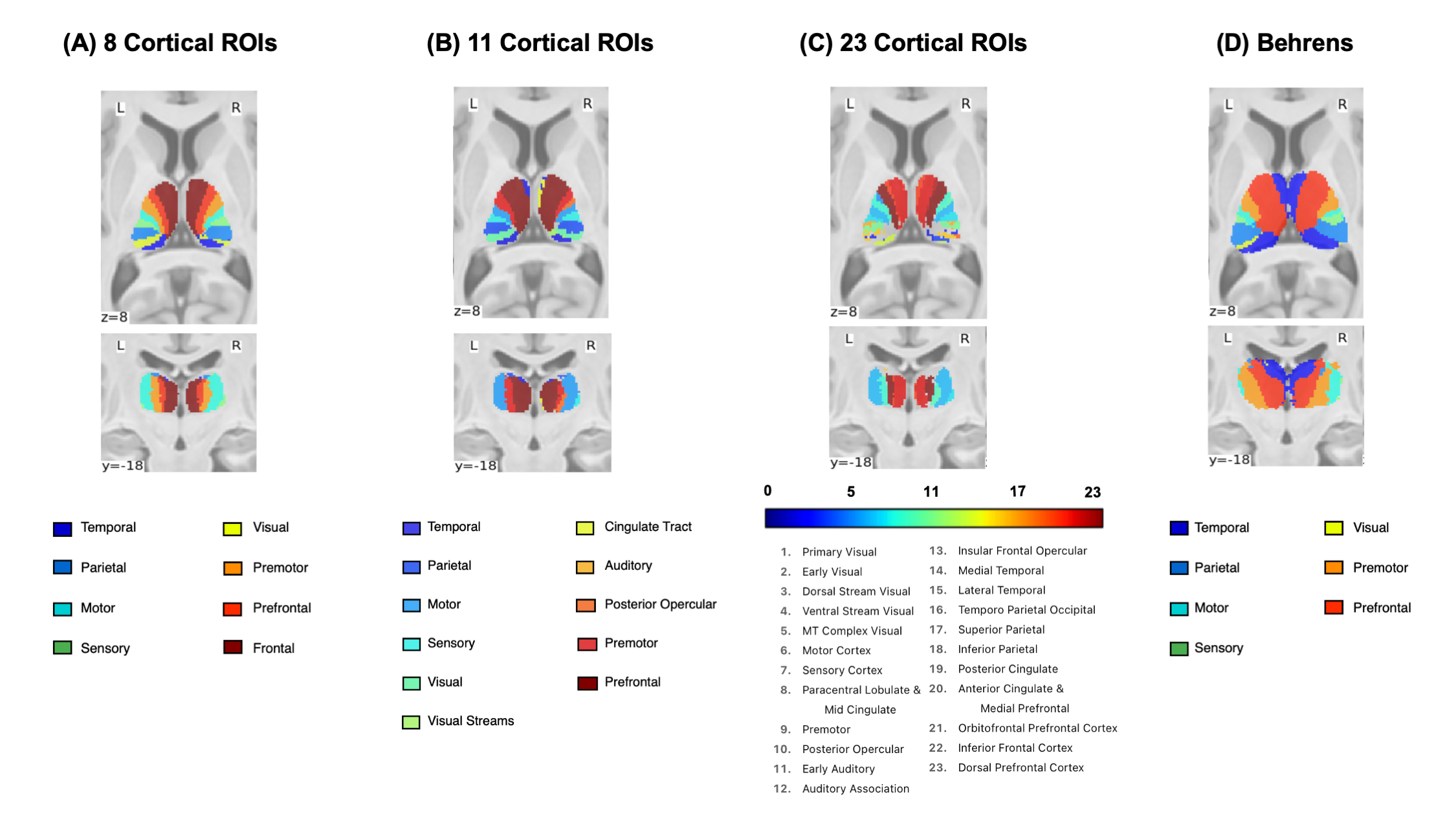

Supplement: S2 Fig — “8 Cortical ROIs” depicts the 8 parcellation at 25% threshold probability. “11 Cortical ROIs” depicts the 11 parcellation at 25% threshold probability. “23 Cortical ROIs” depicts the 23 parcellation at a 25% threshold probability. The Behren parcellation in MNI space is also shown at a 25% threshold probability for comparison. (TIFF) [file pone.0351431.s002.tiff]

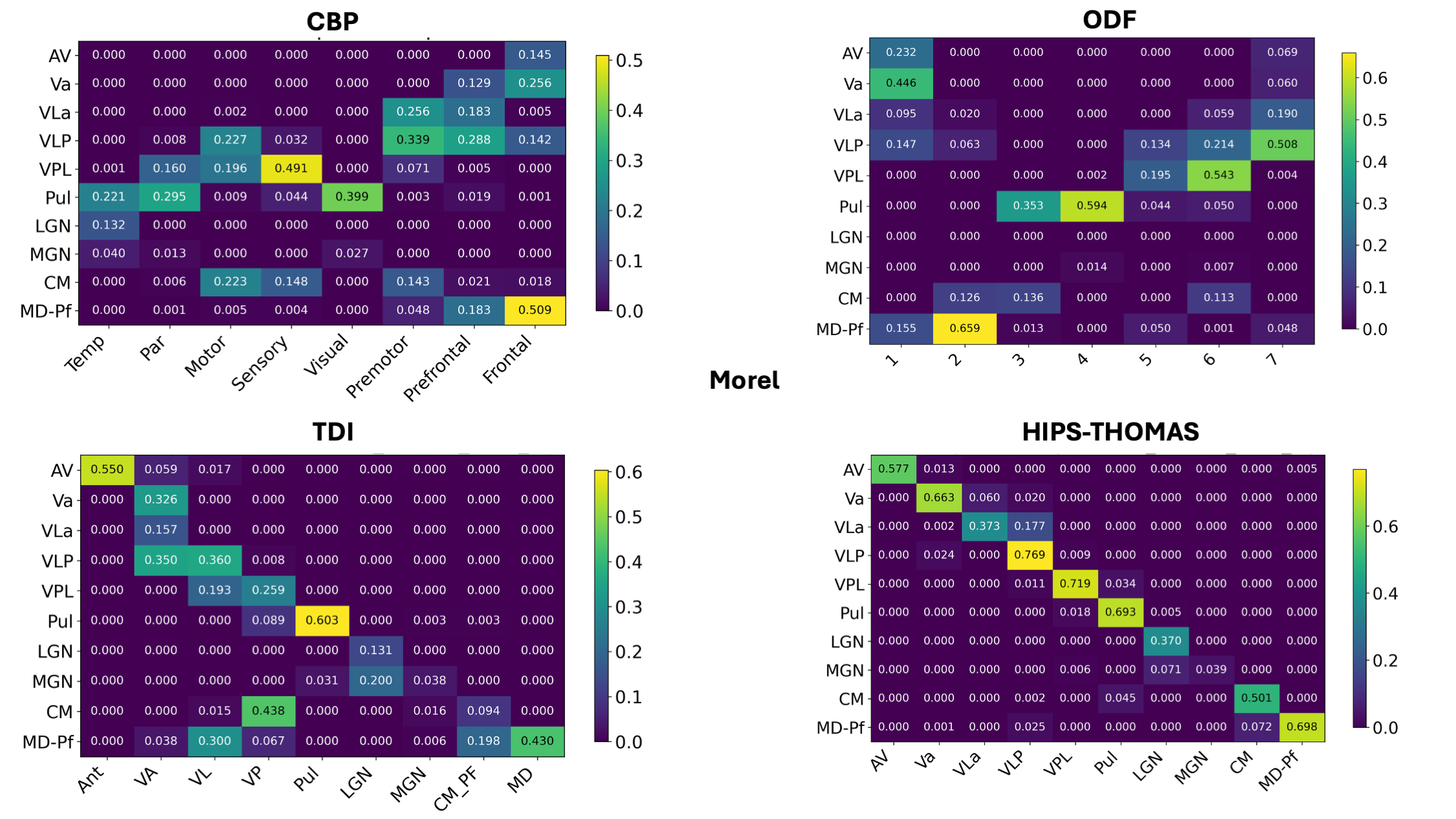

Supplement: S3 Fig — Dice scores were computed between each of the parcels in the Morel atlas and compared against each of the parcels in the 8 parcellation, ODF Clustering, TDI, and Structural (HIPS-THOMAS). Higher Dice signify greater overlap between the two parcels. (TIFF) [file pone.0351431.s004.tiff]

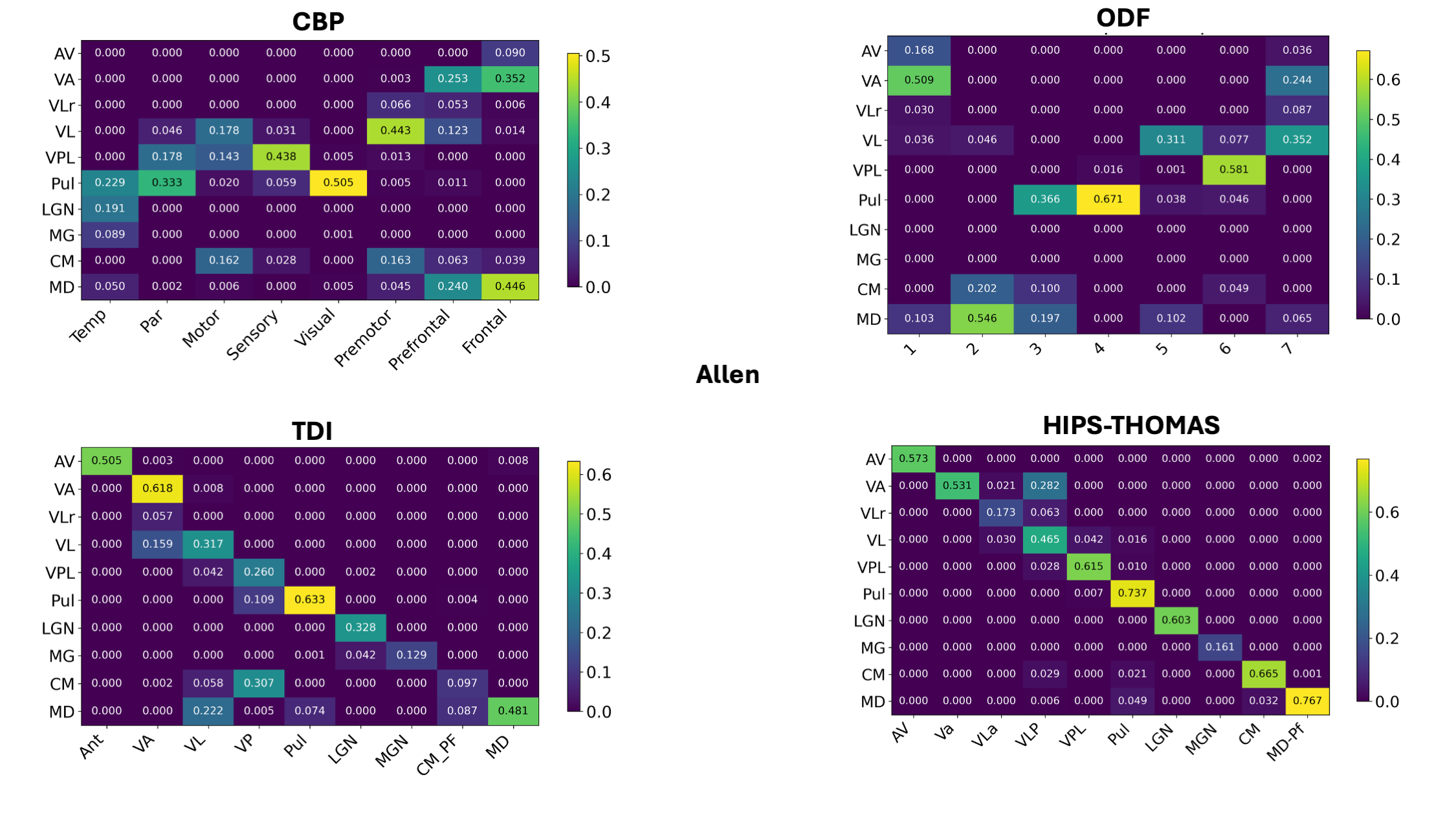

Supplement: S4 Fig — Dice was conducted between each of the parcels in the Allen Human Brain atlas and compared against each of the parcels in the 8 parcellation, ODF Clustering, TDI, and Structural (HIPS-THOMAS). Higher Dice signify greater overlap between the two parcels. (TIFF) [file pone.0351431.s005.tiff]

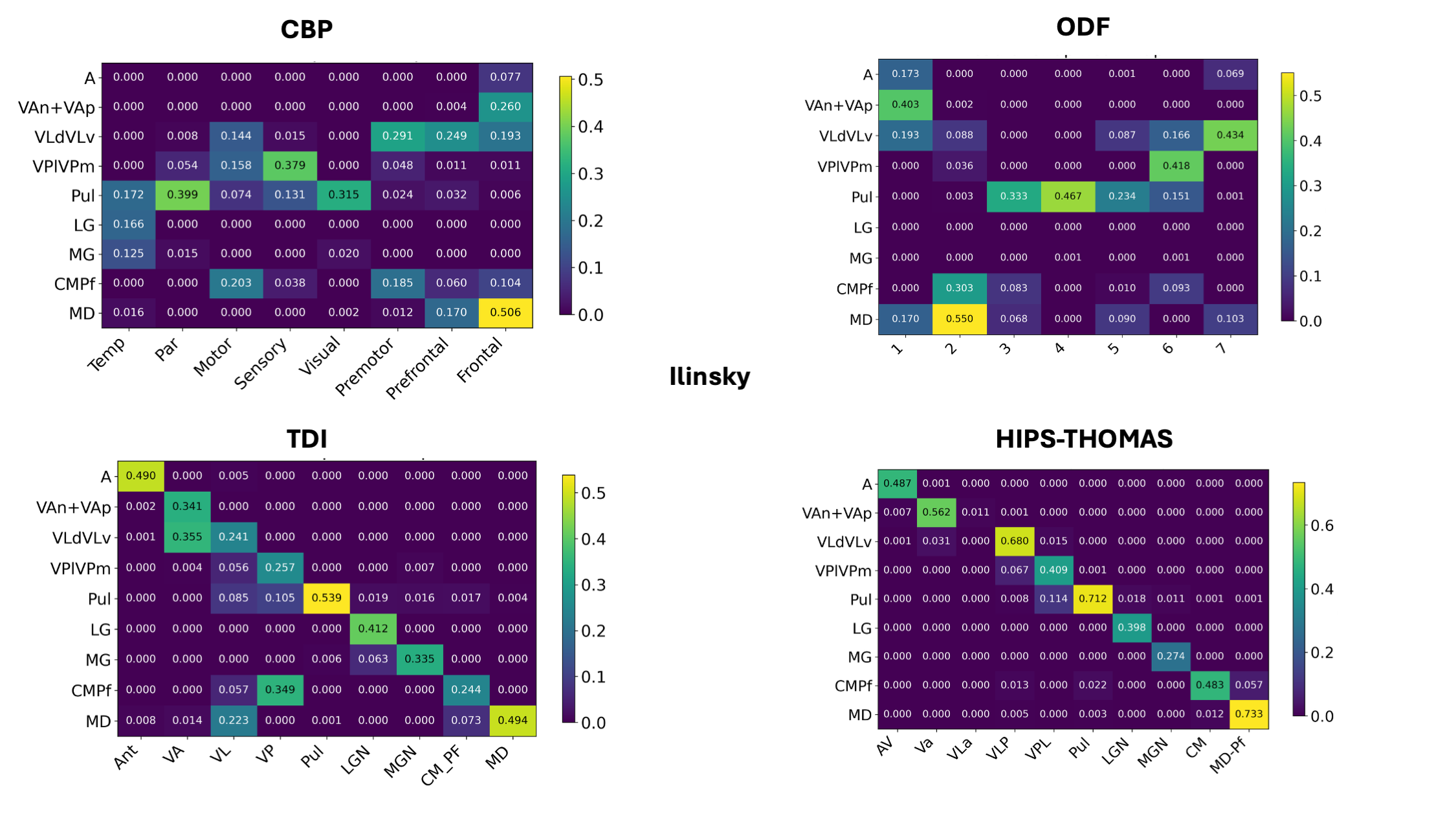

Supplement: S5 Fig — Dice was conducted between each of the parcels in the Ilinsky atlas and compared against each of the parcels in the 8 parcellation, ODF Clustering, TDI, and Structural (HIPS-THOMAS). Higher Dice signify greater overlap between the two parcels. (TIFF) [file pone.0351431.s006.tiff]

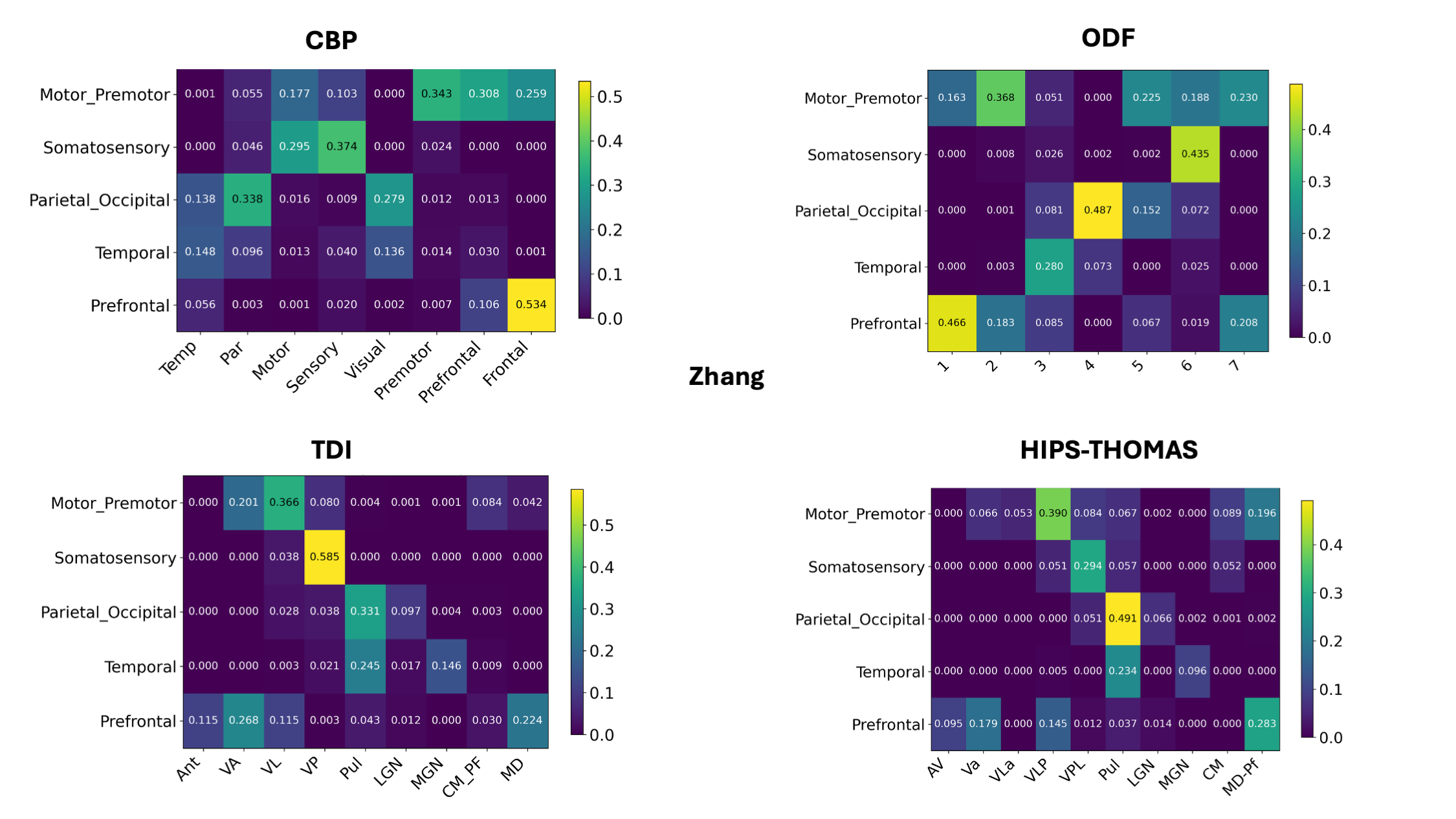

Supplement: S6 Fig — Dice was conducted between each of the parcels in the Zhang atlas and compared against each of the parcels in the 8 parcellation, ODF Clustering, TDI, and Structural (HIPS-THOMAS). Higher Dice signify greater overlap between the two parcels. (TIFF) [file pone.0351431.s007.tiff]

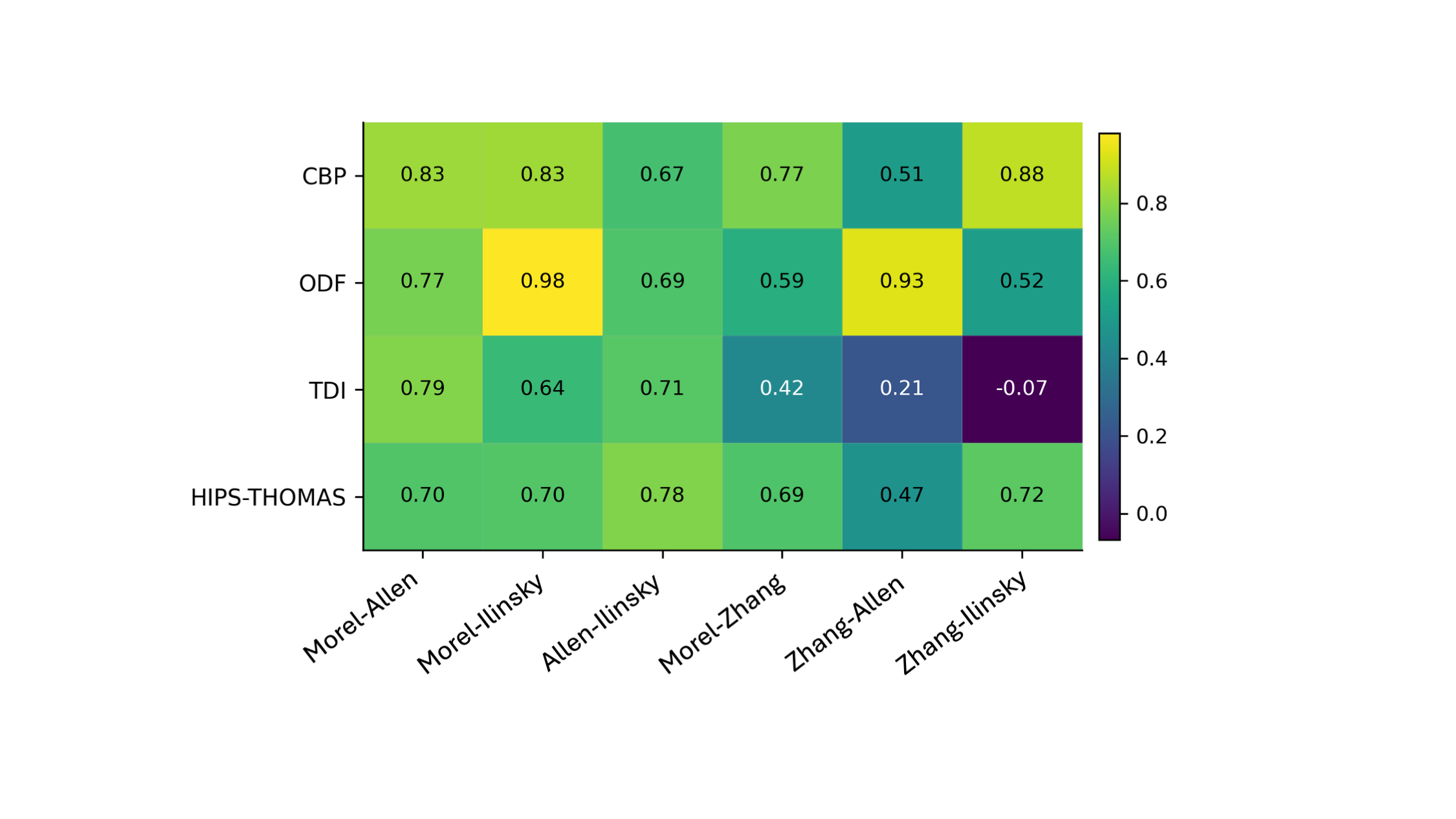

Supplement: S7 Fig — Heatmap showing Pearson correlations (r) of Dice coefficients between atlas pairs for each parcellation method (CBP, ODF, TDI, HIPS-THOMAS). (TIFF) [file pone.0351431.s008.tiff]
