# Supplementary material for: Revisiting the role of structural connectivity-based parcellation in thalamic nuclei segmentation: Benchmarking against recent state-of-the-art methods
Source: PLoS One. 2026 Jun 15;21(6):e0351431. doi: 10.1371/journal.pone.0351431 (PMC13268177; doi:10.1371/journal.pone.0351431)
Supplement: S1 Table — (DOCX) [file pone.0351431.s003.docx]

| **Morel** | **Allen** | **Ilinsky** |
| --- | --- | --- |
| AV | AV | A |
| VA (Vamc + VApc) | VA (VAmc + VApc) | VAn + VAp |
| VLa | VLr |  |
| VLP (VLpd + VLpv + VLp) | VL | VLdVLv |
| VPL (VPLa + VPLp) | VPL (VPLc) | VPlVPm |
| Pul (PulA + Pul + PuL + PuM) | Pul (PulA + PuM + PuL) | Pul |
| LGN (LGNmc + LGNpc) | LGN | LG |
| MGN | MG | MG |
| CM | CM | CMPf |
| MD-Pf (Pf + sPf + MDmc + MDpc) | MD | MD |
